# Surgical Delusions

**Published:** 1884-06

**Authors:** John B. Roberts

**Affiliations:** Professor of Anatomy and Surgery in the Philadelphin Polyclinic


### Surgical Delusions.

AN ABSTRACT OF THE ADDRESS IN SURGERY OF THE MEDICAL SOCIETY FOR THE STATE OF PENNSYLVANIA, FOR 1884.

By John B. Roberts, M. D.

Professor of Anatomy and Surgery in the Philadelphin Polyclinic.

Many surgical theories and procedures have become traditional, and are accepted as true and correct, merely because reverence for antiquity, or careless acceptance, has not questioned their right to be classed as surgical facts. The present age is an incredulous one, and demands accurate investigation of all such claims. The field of investigation is large, for progress has been retarded by the influence of theorizing writers, with monochromatic vision, the example of non-seeing and non-looking devotees of the fetiches of surgical superstition and the convincing effect of a repetition of false statements. I shall select a few topics which have greatly interested me and concerning which I probably differ quite widely from many of you.

### CHLOROFORM ANÆSTHESIA.

Many still cling to the delusion that chloroform is a safe anæsthetic, because they have never seen a patient die from it. Is one man's experience to weigh against the physiological, the experimental, the clinical experience of the whole world? Dare we employ chloroform, instead of ether, when recognized authorities state that in chloroform anæsthesia death occurs without warning in the hands of experienced administrators; when some five hundred deaths have already been reported; when Schiff and Dalton reject it in physiological laboratories, because of its mortality; when the scientific grants committee of the British Medical Association assert that chloroform is a more dangerous anæsthetic than ether.

Adherence to chloroform in the face of such facts is criminal when circumstances permit ether to be obtained. The assertion that it is often impossible to produce anæsthesia with ether is the result of inefficient methods of administration. Ether, if given as chloroform is and should be given, is, in truth, a useless anæsthetic, but given properly it is efficient.

### VALUE OF STYPTICS.

The belief in the necessity of styptics is a delusion less dangerous than that first mentioned, but is given more extended credence. Such agents are seldom, probably never, needed in general surgery to arrest hemorrhage. When ligatures, torsion or acupressure is not demanded, and such is seldom the case unless the artery is as large as the facial, moderate, direct pressure, applied in dressing the wound, is the only hemostatic required. Styptics often do harm, and, as they are not needed, they should be discarded.

FATALITY OF SMALL HEMORRHAGES.

There is much misapprehension about the quantity of blood that a healthy person may lose with impunity. Many who often look with equanimity upon a parturient woman losing a pint of blood from the uterine sinuses would be dismayed at a woman losing half or a quarter that amount during removal of a tumor. While not advocating needless waste of blood, and especially in patients suffering surgical shock, I assert that there is an unnecessary fear of blood spurting from a few insignificant vessels. The largest artery can be controlled by pressure not greater than is used for ringing the electric bell in your hotel. Hence there is always sufficient power in your fingers to obviate fatal hemorrhage until strings can be obtained and applied.

DANGER OF TREPHINING THE SKULL.

The dislike to make exploratory incisions in closed fractures of the skull evinced by some surgeons and the objection of others to trephining, and thus opening the diploic structure in open fractures, are delusions of a most disastrous tendency. To wait until symptoms of cerebral compression or inflammation have supervened is to lose the most favorable opportunity for mechanical relief. Such a Fabian policy is often followed by death. The treatment of open and of closed fractures of the skull should not be looked upon as very different, since, with the present improved methods of dressing wounds, the successful issue depends almost entirely upon the cerebral rather than the cranial phase of the injury. If such fractures as are usually seen in the skull were not in proximity to the brain, the surgeon would consider them almost trivial. The feature of closed fractures that renders them so troublesome is the obscurity that accompanies them. I have for a number of years strongly advocated making closed fractures open ones by means of an exploratory incision, whenever there is a suspicion of the existence of depression or splintering. In open fractures operation to elevate depressed portions and get rid of splinters of the inner table thrust into the membranes should be undertaken rather than avoided. It is better to err on the side of action than that of inaction. Careful manipulation and proper dressings at an early stage are sources of less risk than is incurred by the surgeon who leaves unseen and unsuspected fragments thrust into the membranes or brain.

### OPERATIVE DELAY IN STRANGULATED HERNIA.

A delusion of fatal issue is that leading to postponement of operative interference in strangulated hernia. Repeated attempts at forcible taxis and medical pow-wow-ing with temporizing measures have ended more lives than the use of the knife. Herniotomy done within twelve hours is almost always followed by recovery. Death is to be expected, however, if strangulation has existed for two or three days, and the gut has been bruised by violent manipulation in the endeavor to relieve the contraction by taxis. Moderate taxis under ether, a half day's treatment with cold applications and the internal use of morphia, and a second moderate attempt at taxis, followed, if unsuccessful, by immediate operation, is the sequence to be followed in strangulated hernia. When symptoms of strangulated hernia exist, the slightest fullness and tenderness in one groin over either of the rings is a sufficient localizing indication to warrant operation.

### OPERATIVE DELAY IN ACUTE PHLEGMONOUS INFLAMMATION.

No insane delusion, no Spanish inquisitor ever caused so many hours of excruciating physical torture as the hallucination that acute abscesses and furuncles must not be incised until pointing has occurred. All the world knows that evacuation of imprisoned pus in phlegmonous inflammations means instant relief of the agonizing pain; yet how few of the profession early and freely incise such inflamed tissues unless they first see the yellow pus under the thinned skin or feel the fluctuation of the fluid in the abscess cavity. The pain is caused by the effort of the pus and sloughing tissue to escape. Is it not, then, more rational to make a free incision to-day than to wait till next week? Time and pain are both saved by early incision. If the cut is made before the pus has actually formed, so much the better.

Probably no form of abscess needs early and free incision more imperatively than that under the palmar fascia. Destructive burrowing of pus is prevented by this radical procedure, which also saves the patient many days of poultices and purgatory.

### OPERATIVE DELAY IN MALIGNANT TUMORS.

Much bad surgery results from a delusive postponement of operative interference in malignant diseases. Instant removal is to be practiced in such cases provided the patient is deemed fit to stand the surgical shock.

### NECESSARY FATALITY OF TRAUMATIC TETANUS.

That traumatic tetanus is of necessity fatal is a commonly held opinion. Proper treatment is sometimes neglected because of this belief in its hopelessness. That the prognosis is extremely unfavorable, I admit, but that cases of a severe type recover is undoubted. Chloral hydrate in full doses has given the best results; but I do not propose speaking of therapeutics at this time. I merely wish to impress upon the profession the fact that a fair number of cases of traumatic tetanus have recovered.

### FATALITY OF PERICARDIAL AND CARDIAC WOUNDS.

The prevalent notion of the excessive danger of these wounds is delusional, at least in as far as it teaches that these structures will not brook surgical interference. The pericardial sac should be dealt with exactly as the pleural sac, by aspiration, incision, irrigation and drainage according to the lesion. That simple puncture or aspiration of the heart itself is not accompanied by the expected risk to life has been pretty well shown, though I am not prepared to recommend its general adoption for trivial cardiac conditions.

### SYMMETRY OF NORMAL LIMBS.

Another delusion still existing in many minds is that the extremities are usually of the same length. Clinical and anatomical investigation show that asymmetry in the length of normal limbs is of common occurrence. Therefore, measurements of the legs is cases of fracture are of little value, since it is impossible to know whether it is the femur of a long or a short leg that is the seat of injury.

### USEFULNESS OF TREATING VICIOUS UNION OF FRACTURES.

It is a fact, not sufficiently appreciated, that many cases of deformity, from imperfectly treated fractures of long bones, can be remedied by refracture. Over and over again have I seen cases of grave disability and deformity cured by the application of sufficient force to break the callus uniting the misplaced fragments. Five to six months is not too late to resort to this expedient for correcting what, otherwise, must be a life-long evidence of defective surgical attendance.

There are many other prevalent surgical delusions, such as, that bony union of transverse fractures of the patella and of intracapsular fractures of the femoral neck cannot take place; that chronic purulent discharges from the ear do not need active treatment; that hypermetropia and hypermetropic astigmatism can be properly estimated and corrected, without paralyzing the accommodation; that it is improper to perforate the nasal septum in cases of great deviation; that crooked noses are not amenable to treatment; that corneal operations and cataract extractions should be treated by cotton padding and bandages to the eyes; that fractures should be treated with carved or manufactured splints.

While an earnest advocate of conservative and of reparative surgery, I believe that when operative surgery is demanded it should be aggressive. Delay, indecision and insufficiency impair the value of much surgical work, and are often the legitimate result of a superstitious faith in delusive surgical dogmas

